# Transcriptomics Analysis of Primordium Formation in *Pleurotus eryngii*

**DOI:** 10.3390/genes12121863

**Published:** 2021-11-24

**Authors:** Dou Ye, Fang Du, Yajie Zou, Qingxiu Hu

**Affiliations:** 1 Institute of Agricultural Resources and Regional Planning, Chinese Academy of Agricultural Sciences, Beijing 100081, China; yedou363216@126.com (D.Y.); duf070413@126.com (F.D.); zouyajie@caas.cn (Y.Z.); 2 College of Life Science and Technology, Huazhong Agricultural University, Wuhan 430070, China

**Keywords:** *Pleurotus eryngii*, primordium formation, RNA-seq

## Abstract

Primordium formation is an important stage preceding the growth and development of the *Pleurotus eryngii* fruiting body. However, the molecular mechanisms underlying primordium formation remain unclear. In the present study, comparative transcriptomics was performed between mature mycelia and primordium to analyze the transcriptional properties during primordium formation in *P. eryngii*. A total of 19,655 differentially expressed genes (10,718 upregulated genes and 8937 downregulated genes) were identified. These differentially expressed genes were involved in cell wall degradation, carbohydrate hydrolysis, light perception, and cAMP signal transduction. These results aid further understanding of the transcriptional changes and the molecular processes underlying primordium formation and differentiation, which may lay the foundation for improving the cultivation and quality control of *P. eryngii.*

## 1. Introduction

*P. eryngii* is a delicious edible fungus with excellent nutritional and medicinal value due to being rich in protein, free amino acids, and tocopherols [1,2,3]. Modern pharmacological studies show that *P. eryngii* exhibits a variety of biological activities including antioxidant, anti-hyperlipidemic, anti-tumor, immunoregulatory, and bacteriostatic effects [4,5,6]. Owing to scientific research promoting commercial development, *P. eryngii* cultivation has been successfully transformed from small- to large-scale modes. At present, *P. eryngii* is mainly produced in factories, which brings huge economic benefits. According to a market investigation by the China Edible Fungi Association, the yield of *P. eryngii* has increased rapidly over the past decade, with a total of 1.96 million tons being produced in 2018.

During the growth and development of *P. eryngii*, primordium formation is an important and sensitive stage that indicates the transition of vegetative to reproductive growth. In this stage, the mycelia kink with each other to form a mycelium community, then form a mass of undifferentiated protoplasm tissue, known as the primordium or fruiting body initial [7]. The quality of primordium is directly related to the development of fruiting bodies, and thus greatly influences the yield. Primordium formation is comprehensively regulated by several factors, such as temperature, humidity, light, and genetic properties. Previous studies show that a temperature of 12–16 °C, a 12 h/12 h light/dark cycle, and 85–90% humidity are suitable environmental conditions for promoting the formation and differentiation of primordium. However, the inherent molecular mechanism remains to be explored in depth. Primordium formation involves alterations in the expression of thousands of genes; thus, to improve our understanding of the genetic and molecular mechanisms underlying primordium formation, it is imperative to identify and functionally analyze these related genes.

RNA sequencing (RNA-seq) has increasingly become a useful technology for investigating the molecular mechanisms underlying specific developmental stages of basidiomycetes. Transcriptome analysis of three *Auricularia auricula-judae* strains showed significant differences in biological pathways, including starch and sucrose metabolism, MAPK signaling, biosynthesis of amino acids, and biosynthesis of secondary metabolites, which provided guidance at the molecular level for the breeding of *A. auricular-judae* [8]. Lucía Ramírez et al. [9] used transcriptome sequencing to analyze fresh fruiting bodies and samples of *Pleurotus ostreatus* stored at 4 °C for 7 days. They found that autophagy, RNA metabolism, and protein and carbohydrate turnover increased during cryopreservation, and genes involved in environmental sensing and morphogenesis were significantly expressed in cryopreserved fruiting bodies, improving the understanding of the decay process in postharvest mushrooms. Transcriptome analysis of the three developmental stages of *Wolfiporia cocos* revealed that peroxisome, unsaturation of fatty acids, and degradation pathway were respectively prevalent in three stages [10]. A comparative transcriptomics study between two commercially available *Volvariella volvacea* strains, V9 and V26, identified various candidate genes involved in the fast growth requirement of *V. volvacea*, which provided a valuable resource for strain improvement of this commercially edible mushroom [11]. *A. auricula* has a stronger selenium accumulation ability, and comparative transcriptomics analysis between its mycelia collected from ordinary medium and medium containing 100 μg/g selenium identified the genes and metabolic pathways related to selenium accumulation [12]. Another comparative transcriptomics study between the mycelia and mature fruiting bodies of *Lentinus edodes* found that 1503 genes were upregulated in mycelia and 577 genes were upregulated in mature fruiting bodies. Moreover, the fruiting-body-specific transcripts were significantly enriched in pathways involved in replication, repair, and transcription, helping to clarify the molecular mechanisms underlying the development and beneficial characteristics of mature fruiting bodies [13]. In a further study, the normal and spontaneous albino mutant strains of *Cordyceps militaris* were compared using RNA-seq to elucidate the genes involved in the response to light during pigment synthesis [14]. Another report employed comparative transcriptomics analysis of the immature and mature mycelia of *Pleurotus tuoliensis* to investigate the process of maturation. A total of 451 differentially expressed genes were identified, including those encoding nucleoside diphosphate kinase (NDPK), glycoside hydrolase family proteins, intracellular polygalacturonase, and multifunctional peroxidase [15]. In subsequent work, Du [16] used the same technique to explore key genes related to pileus morphogenesis in *P. eryngii* under different light conditions, and found a potential involvement of genes related to light sensing, signal transduction, cell wall degradation, and melanin production.

The objectives of the present research were to elucidate the differentially expressed genes associated with primordium formation in *P. eryngii* using RNA-seq technology, with a view to uncovering the underlying molecular mechanism and providing a theoretical foundation for the growth and development of *P. eryngii*.

## 2. Materials and Methods

### 2.1. P. eryngii Cultivation and Sample Collection

The *P. eryngii* strain ACCC52611 was generously provided by the Agricultural Culture Collection of China (ACCC). The cultivation substrate consisted of 26.8% corncob, 26.8% sawdust, 11.1% corn flour, 11.1% soybean meal, 22.2% wheat bran, 1% lime, and 1% gypsum. After complete mixing, the substrate was packed into 40 polypropylene cultivation bags (average of 1350 g/bag with a moisture content of 62–65%), sterilized at 121 °C for 2 h, and inoculated with a pure culture of *P. eryngii*. The inoculated bags were randomly arranged in an incubator and maintained at 25 °C in the dark until the cultivation substrate was fully covered by mycelia. Mycelia were collected from three cultivation bags as the control group (A) and frozen at −80 °C until RNA extraction. The remaining bags were placed at 12–14 °C under a 12 h/12 h white light/dark cycle until primordium formed. The primordium was collected from three cultivation bags as the experimental group (B) and frozen at −80 °C until RNA extraction. Each experiment had three biological replicates for the RNA-seq analysis. Thereafter, all bags were subjected to conventional fruiting management.

### 2.2. RNA Extraction, cDNA Library Construction, and Illumina Sequencing

Total RNA was extracted from each sample using a mirVana™ miRNA Isolation Kit (Ambion, Carlsbad, CA, USA) and the mRNA was enriched with Oligo (dT) mRNA magnetic beads. After that, fragmentation buffer was added, the mRNA was interrupted to short fragments, the first strand of cDNA was synthesized by random hexamer primer using the mRNA fragments as templates, and then the second strands of cDNA were synthesized. The double-stranded cDNA was purified, end repaired, and “a-tailed” for adapter ligation. Immediately after, the six cDNA libraries (two treatments with three biological replicates per treatment) were evaluated using an Agilent 2100 Bioanalyzer (Agilent Technologies, Santa Clara, CA, USA). Only samples with an RNA integrity number (RIN) ≥ 7 and 28 S/18 S ≥ 0.7 could be used for subsequent analysis on the Illumina sequencing platform (HiSeq™ 2500, San Diego, CA, USA) and 150 bp paired-end reads were generated.

### 2.3. Bioinformatics Analysis

The raw data containing low-quality reads and the poly-N sequences were processed using the NGS QC Toolkit (http://www.nipgr.res.in/ngsqctoolkit.html, accessed on 6 Semptember 2018) to obtain high-quality clean reads [17]. Subsequently, the clean reads were mapped to the *P. eryngii* genome derived from the JGI transcriptome reference database (https://genome.jgi.doe.gov/Pleery1/download/Pleery1_all_transcripts_20150629.nt.fasta.gz, accessed on 6 Semptember 2018) using hisat2 [18,19]. Then, the transcripts were annotated to the *P. eryngii* annotations derived from the JGI transcriptome annotation database (https://genome.jgi.doe.gov/Pleery1/download/Pleery1_all_genes_20150629.gff.gz, accessed on 3 August 2020). The number of reads per transcript of each sample were obtained using the bowtie2 [20] and eXpress [21] software. The expression levels of the transcripts were calculated using the Fragments Per Kilobase Per Million Reads (FPKM) method:

$$\mathrm{FPKM}=\frac{\;\mathrm{Fragment}\;\mathrm{number}\;\mathrm{of}\;\mathrm{gene}\;A}{\;\mathrm{Fragment}\;\mathrm{number}\;\mathrm{of}\;\mathrm{total}\;\mathrm{genes}\;\times \;\mathrm{length}\;\mathrm{of}\;\mathrm{gene}\;A}$$

Differentially expressed genes were identified using the DESeq R package functions Estimate Size Factors and Nbinom Test [22]. The thresholds for significant differential expression were set as a *p*-value < 0.05 and fold change >2 or <0.5. Hierarchical cluster analysis of differentially expressed genes was performed to explore the gene expression patterns.

Differentially expressed genes were used for Gene Ontology (GO) [23] and Kyoto Encyclopedia of Genes and Genomes (KEGG) [24] enrichment analyses with a corrected *p*-value  ≤  0.01 as a threshold. The *p*-value was corrected with multiple hypothesis testing by calculating false discovery rates (FDRs). The number of differentially expressed genes included in each term was counted and the enrichment significance was tested by hypergeometric distribution. The *p*-value denotes the significance of the GO term or KEGG pathway enrichment in the differentially expressed gene list (threshold for significance, *p* < 0.01). The RNA-seq analysis software is summarized in Table 1.

$$P\left(X=k\right)=\frac{\left(\begin{array}{c} K\\ K \end{array}\right)\left(\begin{array}{c} N-K\\ n-k \end{array}\right)}{\left(\begin{array}{c} N\\ n \end{array}\right)}$$

In the equation above, N is the number of transcripts with GO or KEGG annotations in all transcripts, and *n* is the number of transcripts with GO or KEGG annotations in differentially expressed transcripts in N.

### 2.4. Validation of Transcriptomics Data by RT-qPCR

Our transcriptomics data were validated by quantitative real-time polymerase chain reaction (RT-qPCR). Total RNA was extracted from the mycelium and primordium samples using an RNA Reagent Kit. Each RNA sample was subjected to RNase-free DNase I (TaKaRa, Shiga, Japan) digestion to remove gDNA, and cDNA was subsequently synthesized according to the PrimeScript Genome RT Reagent Kit protocol (Perfect Real Time, TaKaRa, Shiga, Japan). Eight DEGs were randomly selected to verify their relative expression levels. The RT-qPCR primers were designed using Primer 3 and are shown in Table 2. Glyceraldehyde 3-phosphate dehydrogenase (gapdh) was used as the internal control and the relative gene expression levels were calculated using the 2^−ΔΔCT^ method. In addition, the qRT-PCR results were obtained from three biological replicates with three technical replicates for each reaction.

### 2.5. Statistical Analysis

Data were analyzed using Excel 2010 and figures were produced using GraphPad Prism. The differences with a *p*-value < 0.05 were considered statistically significant.

## 3. Results

### 3.1. Analysis of the Morphological Features of P. eryngii

**Figure 1:**
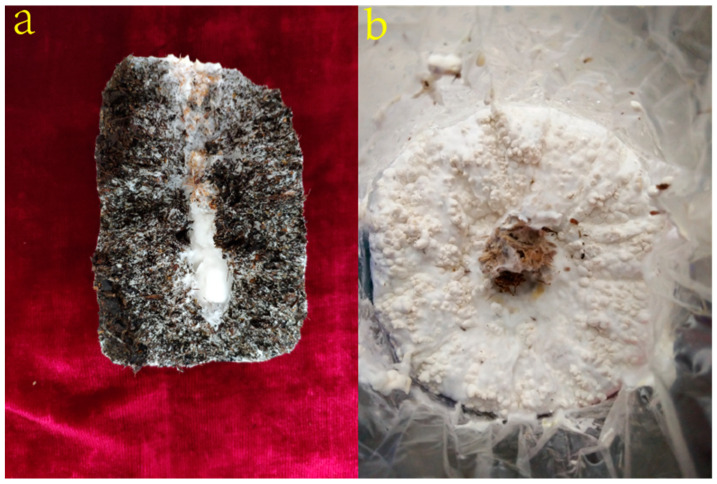
Morphological features of *P. eryngii* at mycelia (**a**) and primordium stages (**b**).

Following incubation at 25 °C for 28 d, the cultivation substrates were fully covered and closely wrapped with mycelia (Figure 1a). Subsequently, the cultivation bags were placed at 12–14 °C under a 12 h/12 h light/dark cycle for 10 d, during which the adhered mycelia kinked and formed a spherical primordium with a white fluffy appearance and hard texture (Figure 1b).

### 3.2. RNA Sequencing

The RNA-seq analysis was performed to explore the molecular mechanisms underlying primordium formation and differentiation. A total of six cDNA libraries were constructed and subjected to Illumina deep sequencing.

The Raw Illumina sequencing data were deposited in GenBank under the BioProject accession PRJNA759001. The clean reads were obtained by processing the raw reads. The percentage of valid bases in all reads was as high as 92%, and the Q30 of all sequences for each library exceeded 90%. More than 90% of the sequences were mapped to the *P. eryngii* reference genome, far exceeding the standard value of 70%. Furthermore, 98% of the sequences were uniquely matched (Table 3). Generally, our sequencing data were of a high quality and could be used for subsequent analysis.

### 3.3. Identification of Differentially Expressed Genes

**Figure 2:**
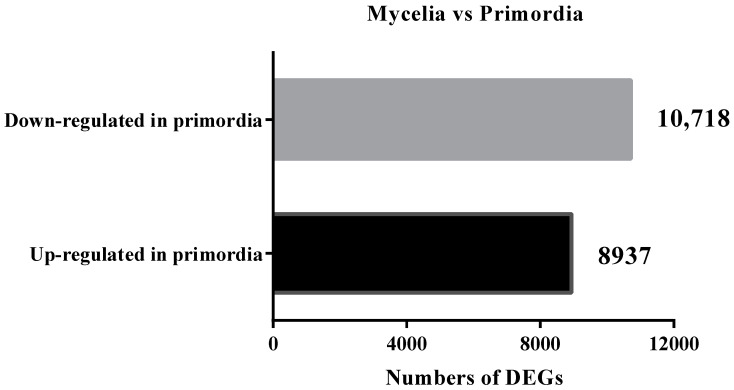
The number of differentially expressed genes during the transformation from mycelia to primordium.

To investigate the changes in mRNA expression between mycelia and primordium, the DEGs (differentially expressed genes) were identified and annotated. Differential expression transcripts were defined as DEGs with adjusted *p*-values < 0.05 and absolute value of |log2 fold change| > 1. Variable splicing makes one gene produce multiple transcripts. Therefore, our statistics would be greater than the number of *P. eryngii* genes predicted on JGI. A total of 19,655 differentially expressed genes were identified (Supplementary Table S1), with 8937 upregulated and 10,718 downregulated in the primordium library, as compared with the mycelium (Figure 2).

### 3.4. Functional Annotation of Differentially Expressed Genes

**Figure 3:**
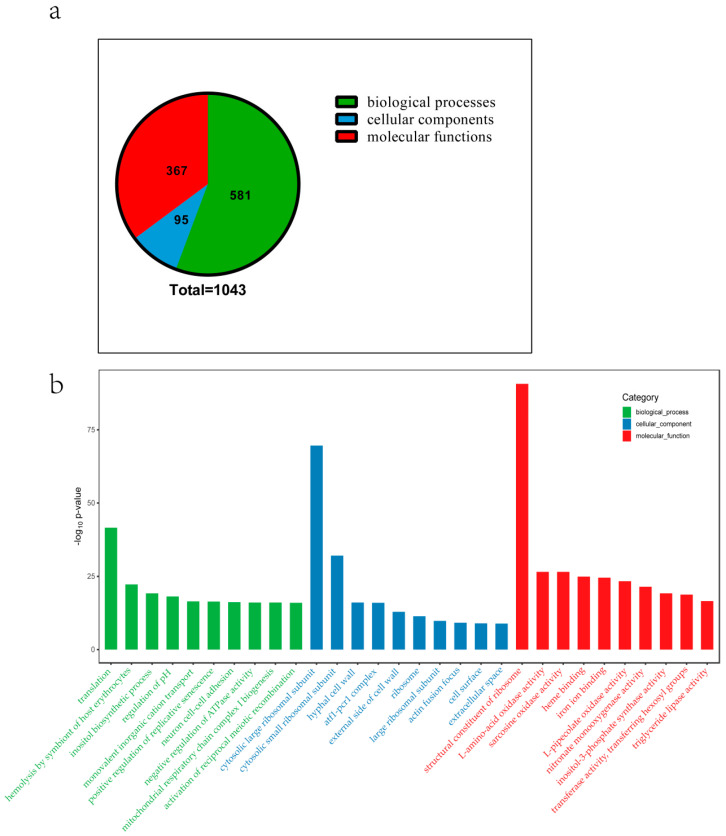
The top 30 GO enrichment terms among DEGs. (**a**) The statistical analysis of GO terms. (**b**) The top 30 GO enrichment terms. The green bars represent biological processes, the red bars represent molecular functions, and the blue bars represent cellular components.

To better understand the function of the DEGs involved in primordium formation, GO enrichment analysis was performed on both upregulated and downregulated DEGs in primordium. The results are summarized in Supplementary Table S2. As shown in Figure 3a, a total of 1043 GO terms were enriched, including 581 biological processes, 95 cellular components, and 367 molecular functions. Figure 3b displays the 30 enriched terms (top 10 terms in each category) (ListHits > 2 and *p* ≤ 0.01). The cellular component terms were related to the cell wall or ribosomes, including the hyphal cell wall (GO:0030446), external side of the cell wall (GO:0010339), and cytosolic small and large ribosomal subunits (GO:0022625, GO:0022627, GO:0005840, GO:0015934). With respect to biological processes, the items enriched in molecular function included structural constituents of the ribosome (GO:0003735), and enzyme activities such as nitronate monooxygenase (GO:0018580) and L-amino acid oxidase (GO:0001716). The formation of *P. eryngii* primordium requires light stimulation. The enriched terms related to light, including response to light stimulus (GO:0009416), response to red light (GO:0010114), red and far-red light phototransduction (GO:0009585), photoreceptor activity (GO:0009881), and photoreceptor inner and outer segments (GO:0001917, GO:0001750), are shown in Table 4. Most belong to biological processes.

**Figure 4:**
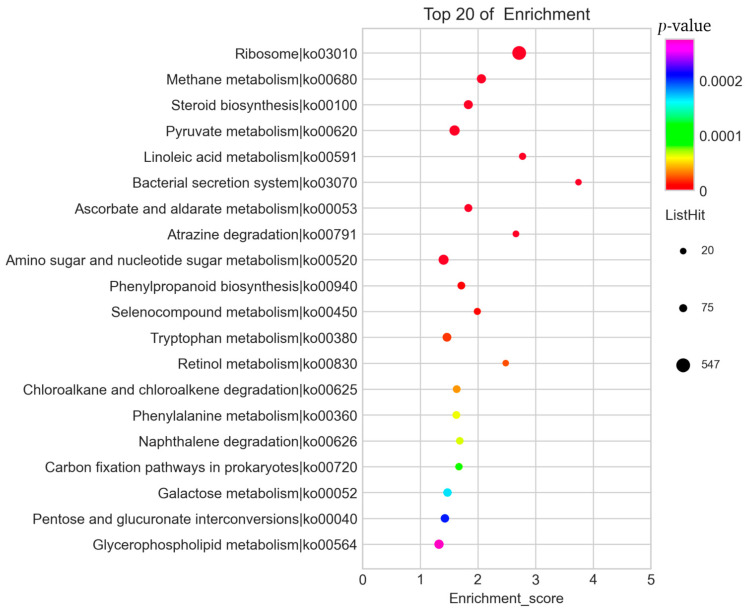
The top 20 KEGG enrichment pathways of DEGs. The *Y*-axis shows the KEGG pathway and the *X*-axis shows the enrichment score. High *p*-values are shown in purple and low *p*-values are shown in red.

We used KEGG analysis to further classify the functions of annotated DEGs, and the results are summarized in Supplementary Table S3. The top 20 KEGG pathways are shown in Figure 4. We observed that most of the DEGs were mainly related to amino acid metabolism, sugar metabolism, ribosomes (ko:03010), and amino and nucleotide sugar metabolism (ko:00520). Therefore, we speculated that these functions might be closely related to the occurrence of primordium.

### 3.5. Differentially Expressed Genes Related to Primordium Formation in P. eryngii

**Figure 5:**
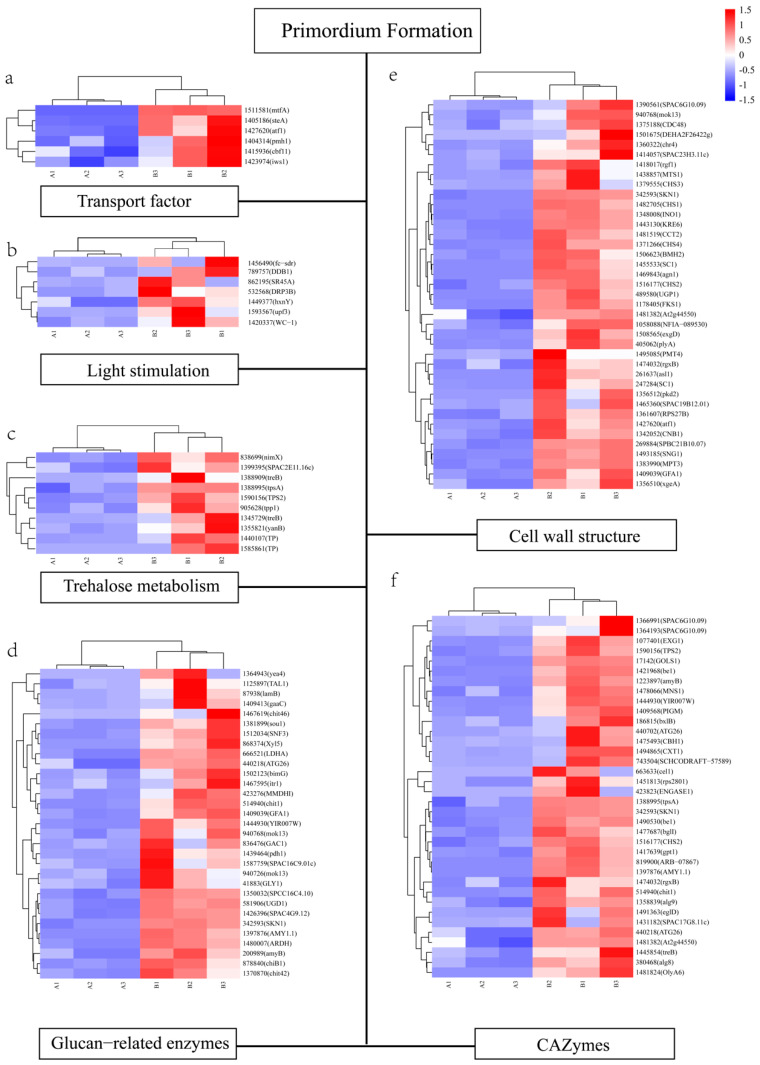
Genes involved in primordium formation of *P. eryngii*. (**a**) Transcription factors. (**b**) DEGs related to light stimulation. (**c**) DEGs related to trehalose metabolism. (**d**) DEGs related to glucan-related enzymes. (**e**) DEGs related to cell wall structure. (**f**) DEGs related to CAZymes. The right side of the heat map indicates the transcript ID in *P. eryngii*, the annotation gene name, and the bottom of the heat map notes the samples. A means mycelium, B means primordium. The gene expression values (FPKMs) were transformed to Z-score values.

Primordium formation is a complex physiological process. We first analyzed genes related to cell wall function (Figure 5). Chitin is a structural component of fungal cell walls. Chitin synthase regulatory factors, including chitin synthase regulatory factor 4 (*CHR4*:1360322), chitin synthase 1 (*CHS1*:1482705), chitin synthase 2 (*CHS2*:1516177), chitin synthase 3 (*CHS3*:1379555), and chitin synthase 4 (*CHS4*:1371266), showed an increasing trend in expression during the primordial stage. In addition, fruiting body protein SC1 (*SC1*:1455533), which is related to the development of the fruiting body, was upregulated by 475-fold in primordium.

Primordium formations need material and energy support, which can be released from the synthesis and catabolism process of carbohydrates. Therefore, the DEGs related to carbohydrate and glycol conjugate metabolism were also analyzed. CAZymes comprise five classes: glycoside transferases (GTs), polysaccharide lyases (PLs), carbohydrate esterases (CEs), glycoside hydrolases (GHs), and auxiliary activities (AAs) [25]. A large number of DEGs encoding CAZymes were determined, including 16 GHs, 13 GTs, and 3 PLs. In addition, dozens of genes encoding glucan-related enzymes exhibited upregulated expression levels during the primordium formation process. 

Primordium formation requires light stimulation; thus, DEGs related to light response were evaluated. White collar 1 (*WC*-1:1420337), a blue light receptor protein, was significantly upregulated during the primordial phase, and is known to participate in the biochemical reaction of protein–chromophore linkage (GO:0018298). This gene also acts as a transcription factor (GO:0043565), which is related to specific DNA binding. Another gene related to protein chromophores, deoxyribodipyrimidine photo-lyase *PHR*(878651), also had significantly upregulated expression in primordium. We also observed that the expression of short-chain dehydrogenase/reductase (*FC-SDR*:1456490) was significantly upregulated in primordium and enriched in terms of the higher light intensity (GO:0042462). 

Finally, we also noticed that multiple genes related to trehalose were differentially expressed during the two developmental periods, including trehalose phosphatase (*TPP1*:905628), neutral trehalase (*TREB*:1345729), and putative α-trehalose-phosphate synthase (SPAC2E11.16c:1399395). In addition, GO analysis uncovered genes that were highly enriched in resistance to external environmental stimuli (trehalose catabolic processes involved in cellular responses to stress, GO:1903134; and trehalose metabolism in response to stress, GO:0070413).

### 3.6. Validation of Transcriptomics Data by RT-qPCR

**Figure 6:**
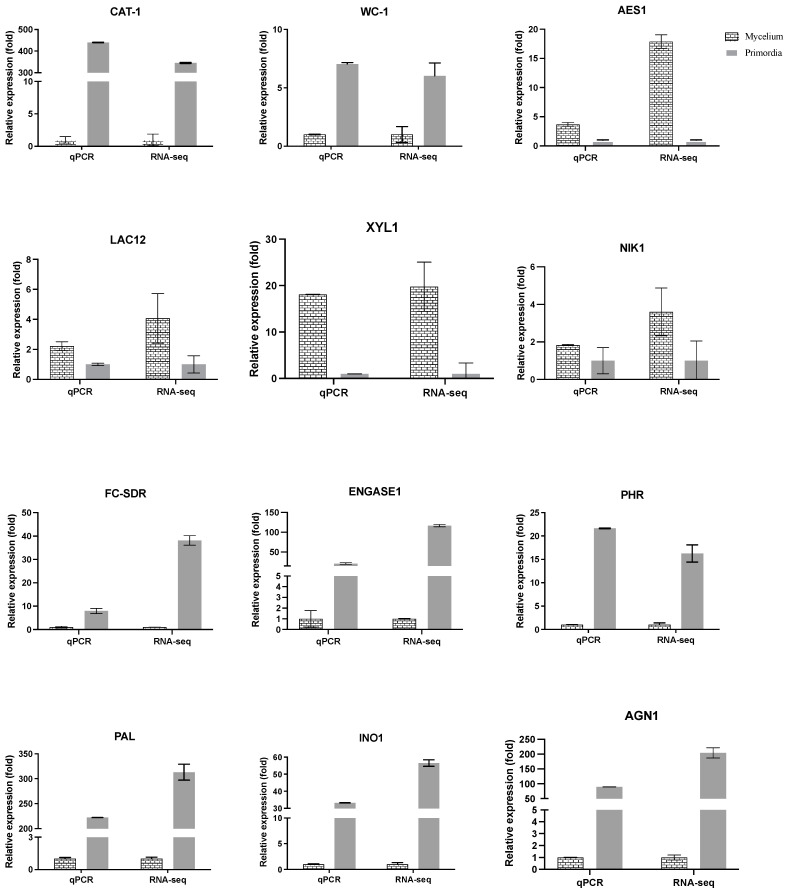
Expression of 12 selected genes as determined by RT-qPCR in comparison with the RNA-seq results. Gapdh expression was used as the internal control. The RT-qPCR values for each gene are expressed as the mean ± SD of three biological replicates.

To confirm the accuracy of the transcriptomics data, eight DEGs were randomly selected for analysis by RT-qPCR to determine their relative expression levels. The RT-qPCR data (Figure 6) are consistent with the transcriptomics data.

## 4. Discussion

The growth and development of *P. eryngii* includes the mycelial, primordial, budding, and fruiting body stages. Primordium formation indicates the transition of vegetative to reproductive growth. Transcriptomics analysis showed that there were 19,655 genes differentially expressed during primordium formation in *P. eryngii*.

Six transcription factors, mtfA (1511581), steA (1405186), atf1 (1427620), cbf11 (1415936), pmh1 (1404314), and iws1 (1423974), were differentially expressed. Among these, atf1 was enriched in two signal transduction cascades, MAPK (GO:0051403) and cAMP-mediated signaling (GO:0043949). The MAPK pathway plays an important role in the physiology and development of fungi [26]. Liu et al. [27] found that many DEGs enriched in the MAPK signaling pathway of *Flammulina velutipes* primordium. In *Coprinopsis cinerea*, the MAPK pathway was determined to be significantly enriched in primordium during the transition from trophic mycelium to primordium [28]. cAMP is a second messenger in both eukaryotes and prokaryotes and plays a key role in fungal development. Lu et al. [29] found that cAMP was highly expressed in the primordium tissue while digging for genes regulating the development of *V. volvacea* and speculated that cAMP was involved in the primordium formation of *V. volvacea*. At the same time, some studies have demonstrated that the cAMP content of *Aspergillus nidulans* and *Dictyostelium* reaches a peak when spores germinate and mature [30,31,32]—that is to say, the cAMP pathway has also been implicated in the development of other fungi. In our study, the upregulation of atf1 expression indicates that cAMP signal transduction and MAPK signaling play regulatory roles in primordium differentiation; however, the specific regulatory mode needs to be further studied.

It has been reported that there is a strong relationship between development and carbohydrate consumption. A total of 32 CAZymes were significantly upregulated in the primordium. We hypothesize that these CAZymes were related to the metabolism and transport of carbohydrates in the primordium, and their high expression levels indicate a large demand for substantial material and energy during primordium formation. However, the downstream effectors of these CAZyme genes remain unknown. A study on *Corynespora cassiicola* leaf fall disease showed that CAZymes are closely related to cell wall degradation; pectin lyases, glucanases, lectin β, and FAD-linked oxidases were effector candidates [33]. In the present study, the genes encoding glucanases and pectin lyases were significantly upregulated, showing that these effector candidates can serve as a breakthrough in molecular mechanism research. 

Many studies have shown that light is a key signaling element for fungal growth and development. Our previous research found that primordium formation and differentiation in *P. eryngii* can only progress normally in the presence of light [34]. Hanbing et al. [35] studied the effect of different light sources on the number of primordia in *Hypsizygus marmoreus* and found the highest number under blue light and the lowest number under red light. The mycelium (or primordium) of *Pleurotus nebrodensis* appears earlier under blue light than under white light; however, formation does not occur under red, green, or yellow light [36]. In the present research, we found that *WC*-1was highly expressed in primordium. *WC*-1 is a blue-light receptor that can respond to light signals and act as a transcription factor, and we speculate that its light response mechanism during primordium formation is similar to that in *Neurospora crassa* [37,38]. In *P. eryngii*, once *WC*-1 receives a light signal, its transcription factor activity is activated, and it participates in the regulation of primordium formation. Photolyase can use light energy to break and repair ultraviolet light products in DNA [39,40]. The upregulated expression of the gene encoding deoxyribodipyrimidine photolyase (phr) indicates the generation of ultraviolet light products, suggesting that white light is a negative signal for the growth and development of *P. eryngii*. This result lays the foundation for a follow-up study of the best light source for primordium differentiation, and phr can be used as a reference indicator for selecting light conditions.

Trehalose is a non-reducing sugar composed of two glucose molecules with 1,1-glycosidic bonds [41], and can be used as a source of carbohydrates, in addition to a typical stress protectant to maintain cell homeostasis [42]. Previous research has shown that trehalose can improve the antifreeze performance of *L. edodes* and *P. ostreatus*, and the growth of mycelia on medium containing trehalose is better than that on medium containing glucose [43]. In this study, the fruiting temperature (14 ± 1 °C) was lower than the hyphae culture temperature (23 ± 1 °C), and the upregulated expression of trehalose-related genes was a stress response of mycelia to adapt to low temperatures.

## 5. Conclusions

Transcriptomics analysis of the mature mycelia and primordium of *P. eryngii* by high-throughput sequencing identified 19,655 differentially expressed genes, including genes encoding chitin related to the cell wall, wc-1 responsive to light, CAZymes related to carbohydrate metabolism and transport, and trehalose responsive to low-temperature environments. These significantly altered genes may be essential to support the formation of primordium; however, the detailed mechanisms need to be studied further. Overall, our data provide a valuable resource for further investigations into the molecular mechanisms underlying the primordium formation of *P. eryngii*.

## Figures and Tables

**Table 1:** RNA-seq analysis software and parameters.

Software	Version	Parameters
NGS QC Toolkit	2.3.3	IlluQC_PRLL.pl N 5-l 70-s 20; TrimmingReads.pl-q 20; AmbiguityFiltering.pl-t5-n 35
hisat2	2.0.5	--RNA-strandness RF--fr
bowtie2	2.2.9	-k30-t
eXpress	1.5.1	--rf-stranded
DESeq	1.18.0	*p*-value < 0.05, |log2FoldChange| > 1

**Table 2:** Primers for RT-qPCR.

Primer	Forward Primer (5′–3′)	Reverse Primer (5′–3′)
*GAPDH*	GCCAACAACTACAACGCAGA	CGCCTGGTACGATAACGAAT
*INO1*	TCCGAAGGACAGATCCTCGT	CGTTGTTACCACCCATCCCA
*WC-1*	CGAATATCGTCCAAGGCGGA	CCCCAAAACTCTCGCTCAGT
*PAL1*	CAACCTAACGCAACAGCAGA	GCCTGCAGACATGGGACTAT
*CAT-1*	CGTGAACACGTACACGCTCT	TCGTCCTTTTCAGGGATGAC
*AGN1*	CCGTTGGGAACAGCTTATGT	GGGTATGAGCCAGTCTGGAA
*PHR*	TAAACCCTTTGGCTCCCGAC	CAGGCGGCTCGATGTATCTT
*AES1*	GATACTGGCCGGGATACAGC	CGGTGATGAGATGCCCTACC
*NIK1*	ATACCGTCAGCCCAGTCTCT	CCGTTCTCCGCTATCTCCAC
*XYL1*	ACGAACGTAAGAGGGCTTGG	AGGACGTTGTACCTTCGCTG
*LAC12*	TGGAACCAACGTGATGCGTA	CGGCAATAAACCTCGAAGCG
*FC-SDR*	AATTTGCAACGGCATCTACC	AATTTGCAACGGCATCTACC
*ENGASE1*	AGGGTGGCTACACAGAAACG	AGGGTGGCTACACAGAAACG

Note: (1) *GAPDH*: glyceraldehyde-phosphate dehydrogenase; (2) *INO1*: Inositol-3-phosphate synthase; (3) *WC-1*: White collar 1 protein; (4) *PAL1*: Phenylalanine ammonia-lyase; (5) *CAT-1*: Catalase-1; (6) *AGN1*: Glucan endo-1,3-α-glucosidase agn1; (7) *PHR*: Deoxyribodipyrimidine photolyase; (8) *AES1*: Acetylesterase. (9) *NIK1*: Histidine protein kinase. (10) *XYL1*: NAD(P)H-dependent D-xylose reductase. (11) *LAC12*: Lactose permease. (12) *FC-SDR*: Short-chain dehydrogenase/reductase. (13) *ENGASE1*: Cytosolic endo-β-N-acetylglucosaminidase 1.

**Table 3:** Summary of the transcript expression levels.

Sample	Raw Reads	Clean Reads	Valid Bases	Q30	GC	Total Mapped	Uniquely Mapped
A1	54,094,426	52,290,698	92.33%	93.88%	53.43%	47,971,181 (91.74%)	47,239,190 (90.34%)
A2	52,370,316	50,312,152	92.34%	93.34%	53.21%	45,726,888 (90.89%)	44,959,009 (89.36%)
A3	53,137,348	51,129,606	92.40%	93.70%	53.29%	46,529,731 (91.00%)	45,731,339 (89.44%)
B1	53,280,494	51,362,248	93.15%	93.67%	53.18%	46,933,670 (91.38%)	46,423,930 (90.39%)
B2	51,919,842	49,990,770	92.77%	93.70%	53.30%	45,633,067 (91.28%)	45,171,712 (90.36%)
B3	52,589,620	50,422,530	92.67%	93.48%	53.35%	46,323,569 (91.87%)	45,859,357 (90.95%)

Note: A: Mycelia sample; B: primordium sample; raw reads: the original number of reads; clean reads: the number of clean reads obtained after filtration; valid bases: percentage of effective bases; Q30: percentage of bases with a Phred value greater than 30 in raw bases out of the total number of bases; GC: the sum of G and C in clean bases as a percentage of the total number of bases; total mapped: statistics on the number of sequences that can be localized to the reference genome; uniquely mapped: statistics on the number of sequences that have a unique comparison position on the reference sequence.

**Table 4:** GO enrichment terms related to light (mycelia vs. primordium).

ID	Term	Category	Samples
GO:2000028	Regulation of photoperiodism	BP	MC
GO:0007602	Phototransduction	BP	MC
GO:0048571	Long-day photoperiodism	BP	PD and MC
GO:0009853	Photorespiration	BP	MC
GO:0042462	Eye photoreceptor cell development	BP	PD
GO:0045494	Photoreceptor cell maintenance	BP	MC
GO:0009585	Red, far-red light phototransduction	BP	PD and MC
GO:0009644	Response to high light intensity	BP	PD and MC
GO:0009416	Response to light stimulus	BP	PD and MC
GO:0009639	Response to red or far-red light	BP	PD and MC
GO:0010114	Response to red light	BP	PD and MC
GO:0001917	Photoreceptor inner segment	CC	PD
GO:0032391	Photoreceptor connecting cilium	CC	MC
GO:0001750	Photoreceptor outer segment	CC	MC
GO:0009881	Photoreceptor activity	MF	PD

Note: BP: biological process; CC: cellular component; MF: molecular function; MC: mycelia; PD: primordium.

## Data Availability

Not applicable.

## Supplementary Materials

The following are available online at https://www.mdpi.com/article/10.3390/genes12121863/s1, Table S1: DEGs Statistics. Table S2: GO enrichment analysis of DEGs. Table S3: KEGG enrichment analysis of DEGs.
